# Novel methods to enhance surgical sperm retrieval: a systematic review

**DOI:** 10.1080/2090598X.2021.1926752

**Published:** 2021-05-18

**Authors:** Eliyahu Kresch, Iakov Efimenko, Daniel Gonzalez, Paul J. Rizk, Ranjith Ramasamy

**Affiliations:** Department of Urology, University of Miami Miller School of Medicine, Miami, FL, USA

**Keywords:** Azoospermia, infertility, micro-TESE, ORBEYE, ultrasonography, artificial intelligence

## Abstract

**Objectives**: To explore the use of novel technologies in sperm retrieval in men with azoospermia due to a production defect.

**Methods**: We performed a Preferred Reporting Items for Systemic Reviews and Meta-Analysis (PRISMA)-compliant systemic literature review for manuscripts focussed on novel sperm-retrieval methods. We identified 30 studies suitable for qualitative analysis.

**Results and Conclusions**: We identified multiple new promising technologies, each with its own distinct set of benefits and limitations, to enhance chances of sperm retrieval; these include the use of multiphoton microscopy, Raman spectroscopy, and full-field optical coherence tomography during a microdissection-testicular sperm extraction procedure. ORBEYE and ultrasonography technologies can also serve to better visualise areas of sperm production. Finally, artificial intelligence technology can play a role in the identification of sperm and, perhaps, better-quality sperm for use with assisted reproduction.

**Abbreviations:** AI: artificial intelligence; ANN: artificial neural network; ART: assisted reproductive technology; 3D: three-dimensional; DNN: deep neural networks; FFOCT: full-field optical coherence tomography; H&E: haematoxylin and eosin; ICSI: intracytoplasmic sperm injection; IVF: *in vitro* fertilisation; MESA: micro-epididymal sperm aspiration; MeSH: Medical Subject Heading; MPM: multiphoton microscopy; (N)OA: (non-)obstructive azoospermia; SCO: Sertoli cell-only syndrome; SRR: sperm retrieval rates; TESA: testicular sperm aspiration; (micro-)TESE: (microdissection-) testicular sperm extraction; (CE)US: (contrast-enhanced) ultrasonography

## Introduction

For couples struggling with infertility, male infertility can be the sole contributing factor in 20–30% of cases and the partial contributing factor in 10–20% [1]. Infertility in males can be attributed to a variety of factors related to the production, transport, or function of sperm. Semen can exhibit decreased concentration of sperm (oligospermia), decreased motility of sperm (asthenospermia), decreased morphology of sperm (teratospermia), and finally, absent sperm in the ejaculate (azoospermia) [2]. In cases of azoospermia or absent ejaculate altogether, treatments can be focussed on obtaining adequate sperm samples for use in assisted reproductive technology (ART). Traditionally, these treatments have included vasal, epididymal, or testicular aspiration. In the present review, we will discuss recent advances in surgical sperm retrieval for non-obstructive azoospermia (NOA), focussing primarily on microdissection-testicular sperm extraction (micro-TESE)

The joint American Society of Reproductive Medicine (ASRM) and AUA 2020 male infertility guidelines highlight that for men with NOA undergoing sperm retrieval, micro-TESE should be performed [3]. Furthermore, in a meta-analysis of sperm retrieval rates (SRR) for men with NOA, micro-TESE was found to result in successful extraction 1.5-times more often than non-microsurgical TESE. Additionally, TESE was twice as likely to succeed when compared to testicular sperm aspiration (TESA) [4].

The efficacy of micro-TESE is limited by the ability of the surgeon to identify seminiferous tubules containing spermatozoa, especially with patients who have Sertoli cell-only syndrome (SCO). When micro-TESE is employed in patients with NOA, the SRR are reported to range from 43–63% [5–7]. The seminiferous tubules are currently evaluated by subjective assessment of their size and opacity, utilising the operating light microscope. Although micro-TESE has become first-line in sperm retrieval in men with NOA, there are some challenges with the procedure, including difficulty differentiating between seminiferous tubules with normal and abnormal spermatogenesis, as well as extensive tissue dissection that can sometimes lead to lifelong testosterone deficiency [7]. Some of the latest advances on the horizon, such as multiphoton microscopy (MPM), Raman spectroscopy (RS), and full-field optical coherence tomography (FFOCT) have demonstrated the potential to better identify areas of spermatogenesis and improve sperm extraction success [8]. We will also elaborate on the use of ORBEYE (a novel 4 K three-dimensional [3D] surgical exoscope), ultrasonography (US), and artificial intelligence (AI) technology to maximise success with both identification of sperm, as well as strategies to enhance sperm selection for ART.

## Methods

The search strategy was conducted according to a modified Preferred Reporting Items for Systematic Reviews and Meta-Analyses (PRISMA) guidelines [9] (Table 1). A literature search was performed using PubMed, the Medical Literature Analysis and Retrieval System Online (MEDLINE), the Excerpta Medica dataBASE (EMBASE), and Cochrane electronic databases to identify studies investigating novel sperm retrieval and identification methods utilising micro-TESE, US, and AI for the years 1999–2020. The search was executed using the following keywords: ‘novel’, ‘surgical sperm retrieval’, ‘sperm retrieval’, ‘mTESE’, ‘microdissection testicular sperm extraction’, ‘on obstructive azoospermia,’ ‘NOA’ ‘Azoospermia’, ‘ORBEye’, ‘Artificial intelligence’ and ‘sperm identification’. Medical Subject Heading (MeSH) phrases included: (‘Artificial Intelligence’[MeSH]) AND ‘Azoospermia/therapy*’[MeSH], AND (‘sperm retrieval’[MeSH]) AND (‘1999ʹ[Date-Publication]: ‘2020ʹ[Date – Publication]).

**Table 1.** PRISMA 2009 checklist

Section/topic	#	Checklist item	Reported on page #
TITLE			
Title	1	Identify the report as a systematic review, meta-analysis, or both.	1
ABSTRACT			
Structured summary	2	Provide a structured summary including, as applicable: background; objectives; data sources; study eligibility criteria, participants, and interventions; study appraisal and synthesis methods; results; limitations; conclusions and implications of key findings; systematic review registration number.	2
INTRODUCTION			
Rationale	3	Describe the rationale for the review in the context of what is already known.	2
Objectives	4	Provide an explicit statement of questions being addressed with reference to participants, interventions, comparisons, outcomes, and study design (PICOS).	2
METHODS			
Protocol and registration	5	Indicate if a review protocol exists, if and where it can be accessed (e.g. Web address), and, if available, provide registration information including registration number.	3
Eligibility criteria	6	Specify study characteristics (e.g. PICOS, length of follow-up) and report characteristics (e.g. years considered, language, publication status) used as criteria for eligibility, giving rationale.	3
Information sources	7	Describe all information sources (e.g. databases with dates of coverage, contact with study authors to identify additional studies) in the search and date last searched.	3
Search	8	Present full electronic search strategy for at least one database, including any limits used, such that it could be repeated.	3
Study selection	9	State the process for selecting studies (i.e. screening, eligibility, included in systematic review, and, if applicable, included in the meta-analysis).	3
Data collection process	10	Describe method of data extraction from reports (e.g. piloted forms, independently, in duplicate) and any processes for obtaining and confirming data from investigators.	3
Data items	11	List and define all variables for which data were sought (e.g. PICOS, funding sources) and any assumptions and simplifications made.	3
Risk of bias in individual studies	12	Describe methods used for assessing risk of bias of individual studies (including specification of whether this was done at the study or outcome level), and how this information is to be used in any data synthesis.	3
Summary measures	13	State the principal summary measures (e.g. risk ratio, difference in means).	3
Synthesis of results	14	Describe the methods of handling data and combining results of studies, if done, including measures of consistency (e.g. I^2^) for each meta-analysis.	-
Section/topic	#	Checklist item	Reported on page #
Risk of bias across studies	15	Specify any assessment of risk of bias that may affect the cumulative evidence (e.g. publication bias, selective reporting within studies).	3
Additional analyses	16	Describe methods of additional analyses (e.g. sensitivity or subgroup analyses, meta-regression), if done, indicating which were pre-specified.	-
RESULTS			
Study selection	17	Give numbers of studies screened, assessed for eligibility, and included in the review, with reasons for exclusions at each stage, ideally with a flow diagram.	Figure 1
Study characteristics	18	For each study, present characteristics for which data were extracted (e.g. study size, PICOS, follow-up period) and provide the citations.	3
Risk of bias within studies	19	Present data on risk of bias of each study and, if available, any outcome level assessment (see item 12).	-
Results of individual studies	20	For all outcomes considered (benefits or harms), present, for each study: (a) simple summary data for each intervention group (b) effect estimates and confidence intervals, ideally with a forest plot.	-
Synthesis of results	21	Present results of each meta-analysis done, including confidence intervals and measures of consistency.	-
Risk of bias across studies	22	Present results of any assessment of risk of bias across studies (see Item 15).	-
Additional analysis	23	Give results of additional analyses, if done (e.g. sensitivity or subgroup analyses, meta-regression [see Item 16]).	-
DISCUSSION			
Summary of evidence	24	Summarize the main findings including the strength of evidence for each main outcome; consider their relevance to key groups (e.g. healthcare providers, users, and policy makers).	10
Limitations	25	Discuss limitations at study and outcome level (e.g. risk of bias), and at review-level (e.g. incomplete retrieval of identified research, reporting bias).	10
Conclusions	26	Provide a general interpretation of the results in the context of other evidence, and implications for future research.	10
FUNDING			
Funding	27	Describe sources of funding for the systematic review and other support (e.g. supply of data); role of funders for the systematic review.	12

*From*: Moher D, Liberati A, Tetzlaff J Preferred Reporting Items for Systematic Reviews and Meta-Analyses: the PRISMA Statement. PLoS Med 2009;6: e1000097. DOI:10.1371/journal.pmed1000097

For more information, visit: www.prisma-statement.org.

**Figure 1.**
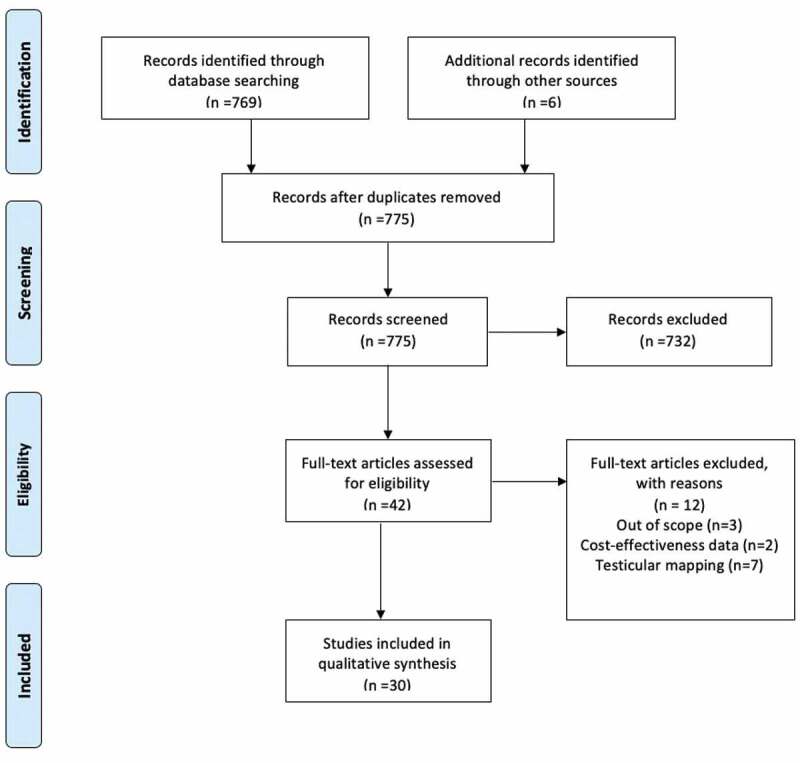
PRISMA flowchart of screening and selection procedure

## Results

The search identified 769 records; an additional five reports were identified via searching the references of relevant manuscripts, and recent published abstracts were considered for inclusion. Inclusion criteria for technologies used to enhance the micro-TESE technique included the following: manuscripts describing the use of novel technologies in the setting of sperm extraction with mice or human subjects. Studies that evaluated solely the theory of these technologies, and not test efficacy on human or mice subjects were excluded. Manuscripts describing novel technologies specific to procedures other than sperm extraction, and manuscripts in languages other than English were excluded.

Following the literature search and application of exclusion criteria, 30 studies were included in the final qualitative synthesis (Figure 1). The final studies were divided up as follows:

The section on MPM included three studies, two on *ex vivo* human tissue and one in rats.

The section on RS included studies on mitochondrial tissue of human and rat spermatozoa.

The FFOCT section included three new studies, which focussed solely on analysing murine tissue.

Due to the novelty in the following technologies some studies were included that did not directly pertain to the field of urology or sperm retrieval as the current literature is limited. However, we felt that in order to gain a full understanding of the technologies these studies should be taken into account.

The ORBEYE section included two studies in the final manuscript. One study compared the ORBEYE with traditional operating microscopes in surgeries with human patients. The other study focussed on results from vasectomy reversal procedures done on rats.

The US section included 12 studies in the final manuscript, 11 of which applied directly to the field of Urology and one from other subspecialties. Of the 12 studies, 11 explored US as a tool for aiding sperm retrieval in human patients. The remaining study focussed on results focussed on global use of US on human patients.

The AI section included eight studies in the final manuscript; six of which applied directly to the field of Urology and two from other fields. Five of the cited studies compared the use of AI with data from traditional methods of extraction with human patients. One study involved identification of spermatozoa parameters in domestic cats. The remaining study focussed on general knowledge regarding AI.

## Discussion

### Multiphoton microscopy

**Figure 2.**
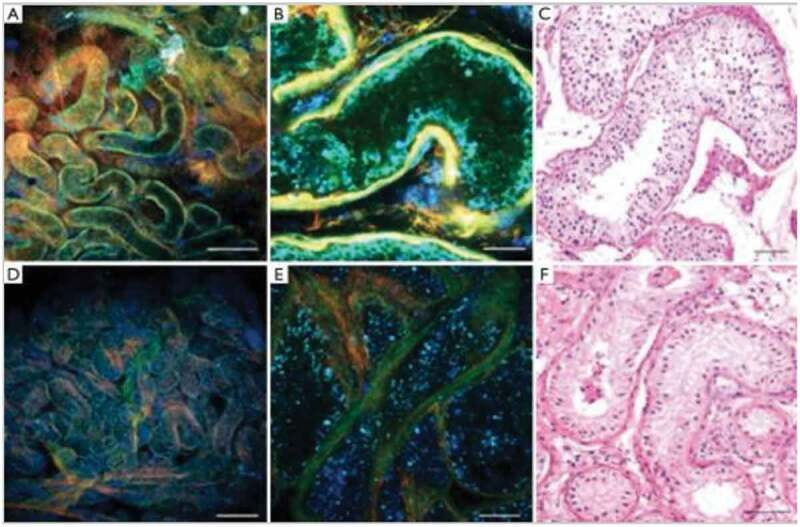
Seminiferous tubular histology patterns imaged by MPM at low (a and d) and high (b and e) magnification compared to high magnification stained tissue (c and f). Normal spermatogenesis is shown by green areas in A to C and seminiferous tubules with SCO pathology is shown by blue areas in D to F. H&E (c and f). Scale bar represents 500 μm (a and d) and 80 μm (b,c,e,f). Permission for reproduction obtained from Elsevier Publishing, Ramasamy et al. [10]

MPM has several advantages over other forms of microscopy. MPM uses a near infrared femtosecond pulsed laser with two or three low-energy photons to produce the excitation of intrinsic fluorophores causing autofluorescence (Figure 2) [10]. The MPM’s near infrared light passes relatively unhindered through tissue, without the need to use additional dyes (that can damage sperm), and enables deeper imaging than other imaging techniques. The penetration depth for MPM is up to 400 µm below the surface, allowing the surgeon to image the lumina of seminiferous tubules. The underlying tissue is optically sectioned, allowing for real-time high-resolution images without the need for physical extraction [10]. Additionally, MPM-guided testis biopsies could potentially prevent the risk of iatrogenic male hypogonadism by optimising the ability to identify only sperm containing tubules and prevent loss of Leydig cells in interstitial testicular tissue [11].

The photons can be combined and scattered in non-centrosymmetric tissue, such as collagen and oriented microtubules, allowing for the visualisation of peritubular fibrosis, typically present in testes with severely defective spermatogenesis [12]. In a pilot study by Najari et al. [13], MPM demonstrated a 92% concordance rate of diagnosis compared to haematoxylin and eosin (H&E) staining in men with NOA, and accurately differentiated normal from abnormal spermatogenesis in human testicular tissue. That study validates the potential impact MPM could have on sperm retrieval in men with NOA; however, there are still some impediments.

Although MPM has shown promising results in enhancing the identification of seminiferous tubules with sperm, further studies must be done to ensure safety of the laser intensity and ethical issues regarding assisted reproduction. MPM safety concerns include thermal and non-linear damage to DNA that can potentially induce genetic abnormalities in gametes used for *in vitro* fertilisation (IVF). Although rodent models showed minimal phototoxicity, these findings have yet to be validated in a human model.

### Raman spectroscopy

**Figure 3.**
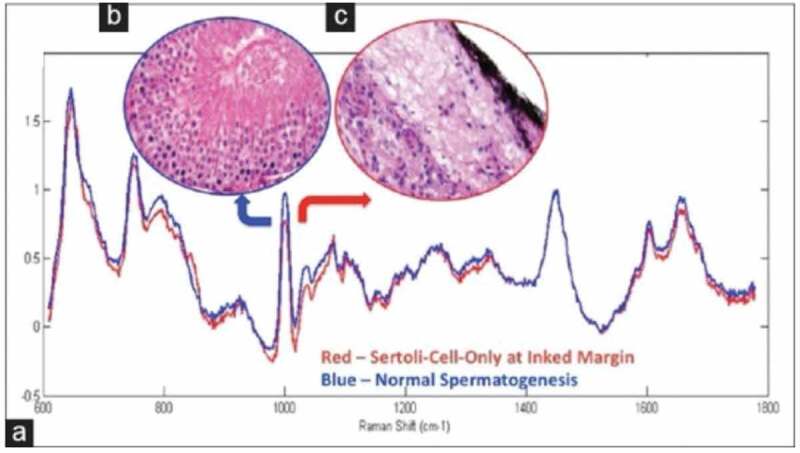
(a) Mean processed spectra for SCO (red curve) and active spermatogenesis (blue curve) with 1000 and 1690 cm^−1^ discriminatory Raman peak intensity, respectively. (b) Representative testicular biopsy shows active spermatogenesis. (c) Representative testicular biopsy shows SCO. (b and c) H&E, reduced from ×200. Permission for reproduction obtained from Elsevier Publishing, Osterberg et al. [16]

RS is a laser-based, optically label-free probe derived from the principle of inelastic scattering from molecular vibrations. RS utilises the molecular fingerprints of different tissues and transforms the biochemical information into a characteristic Raman spectrum [14]. In reproductive medicine, RS was first utilised to evaluate sperm DNA integrity and to distinguish between spermatozoa that could bind to the zona pellucida [15]. This technique was 96% sensitive and 100% specific in distinguishing the presence of spermatogenesis in rat models with SCO histology (Figure 3) [16]. Given that the sensitivity and specificity of RS are greater than any other techniques discussed thus far, RS-guided micro-TESE could have the potential to improve SRR [16]. While this technique is non-invasive and non-destructive, the overall safety of this laser-based technique needs to be assessed in human models. While this is a real-time analytical tool, each analysis takes ~2 min and results can be skewed by light pollution [17].

### Full-field optical coherence tomography

**Figure 4.**
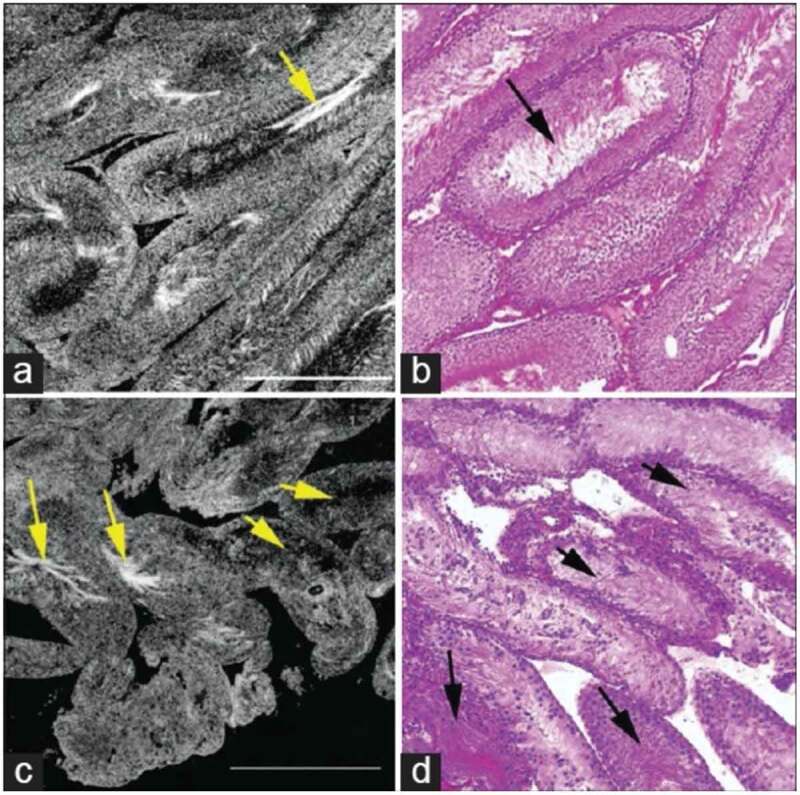
Comparative FFOCT and H&E-stained histology. (a) Testis of a normal rat shows seminiferous tubules with relatively uniform size and shape, (b) H&E histology stain of the same specimen. Arrows point to the sperm within the tubule lumen. (c) Seminiferous tubules in the testis of a rat treated with busulfan, showing thinner tubules and a greater degree of heterogeneity in size and shape with ~10% normal spermatogenesis. (d) H&E staining of the same specimen. Field of view in each panel: 1 mm^2^ Permission granted under the creative commons attribution license, Ramasamy et al. [19]

FFOCT is a technique that uses a simple tungsten halogen lamp and is based on the principle of white-light interference microscopy to produce high-resolution images of unprocessed and unstained tissue [18]. Some of the advantages of FFOCT are the speed (1 frame/s) and ease with which images can be obtained from relatively large areas of tissue. One specific advantage that the FFOCT harbours over MPM and RS is the use of very safe incident light coming from a 150-W halogen lamp, making it ideal for IVF with intracytoplasmic sperm injection (ICSI), as it decreases the potential of thermal DNA damage in extracted sperm. In a pilot animal study, Ramasamy et al. [19] demonstrated that FFOCT successfully distinguished between tubules with and without spermatogenesis, by imaging testicular specimens from a busulfan-treated rodent model (Figure 4) [19]. Normal adult rats exhibited tubules with uniform size and shape (mean [SD] diameter 328 [11] µm), while busulfan-treated rats showed marked heterogeneity in tubular size and shape (mean [SD] diameter 178 [35] µm), with only 10% containing sperm within the lumen. FFOCT defined spermatogenesis as the presence of a bright signal, which emanates from the unique microtubular structure in sperm tails. Unlike MPM, FFOCT has considerable limitations, including the absence of cellular details, limited depth of imaging below the specimen surface, and the fact that this system can only image *ex vivo* specimens [19,20]. Unfortunately, further studies have not been carried out in human testicular tissue, and it is unknown if whether FFOCT displays the same efficacy for identifying spermatogenesis given the complex cellular microtubular structure.

### ORBEYE

**Figure 5.**
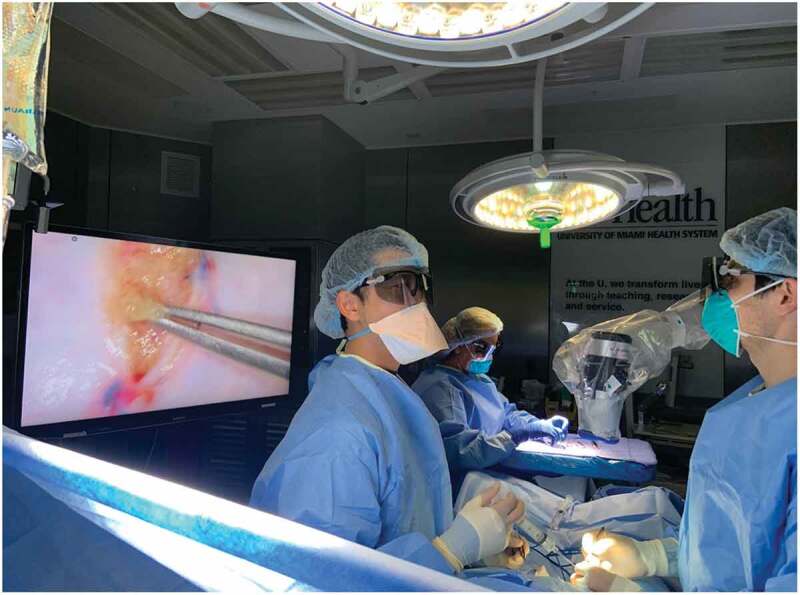
Operating room setup with the ORBEYE™ surgical microscope. The microscope coming from the surgeon’s left-frontal side is held over the surgical field resulting in no obstacle between the surgeon and the monitor. The operator, the assistant and the entire operating staff using 3D glasses have the same view as the operator

The ORBEYE 4 K 3D microscope is a surgical exoscope or ‘camera’ that can be used to enhance urological microsurgical procedures such as micro-epididymal sperm aspiration (MESA) or micro-TESE [21]. It consists of two Sony 4 K (4096 × 2160 pixels) Exmor R CMOS image sensors, which help provide high sensitivity, low noise, and a wide colour range image. The exoscope is placed over the surgical field and the image is projected on to two 140-cm (55-inch) monitors that allow for active 3D viewing with lightweight passive light 3D glasses (Figure 5).

Historically, surgeries done with state-of-the-art microscopes allowed for high magnification and detailed views of the surgical field. However, they did necessitate frequent repositioning due to the shallow depth of field and required surgeons to constantly fixate their eyes into the eyepieces of the microscope [22]. The ORBEYE overcomes both difficulties with the use of wider fields of view and longer depths of field. The use of video monitors eliminates the need for eyepieces, which forces surgeons to hold uncomfortable postures at awkward angles. It also allows other members of the operative team, including staff and students who may not be participating in surgery, to be able to learn from and follow the surgical steps in as well, by wearing the 3D glasses [23].

The ORBEYE is already widely used in surgical fields with microsurgical subspecialties. It was first reviewed to address its advantages and disadvantages in micro-neurosurgery in 2018 [24]. In urology, ORBEYE was compared to the traditional operating microscope for vasectomy reversal in a prospective randomised controlled animal trial on rats in 2019 [25]. The study concluded that there was no difference with respect to patency, operating time, or granuloma formation. Another study analysed the differences in operating time and surgeon fatigue for urological microsurgery, including MESA and micro-TESE, between the two scopes [21]. Although the difference was not statistically significant (*P* = 0.092), operating times for varicocelectomies appeared to be shorter with ORBEYE than traditional microscopes. The logistics of transport, draping, and operating seem to be advantageous with the ORBEYE, which can be attributed to its manoeuvrability, compactness, and simple plug-and-play interface. The ORBEYE also appears to have an ergonomic advantage over traditional microscopes, allowing surgeons a more natural heads-up posture. This is especially important in urology, with a relatively large amount of time spent by surgeons in high-risk neck positions [26]. Similar outcomes were reported in neurosurgery and vascular surgery [24,27]. A potential disadvantage comes with the cost of the ORBEYE, which runs in the range of 400,000 USD (American dollars), about twice as much as traditional operating microscopes. Another potential disadvantage lies in operating from the point of view of surgical assistants, as a rotated view of the surgical field will be projected on to the monitor from the assistant’s position.

### Ultrasonography

Due to the invasive nature of biopsies and poor predictive ability of clinical characteristics, testicular imaging of men with NOA is an area of great interest. US has been investigated as a non-invasive and widely accessible method for evaluating a patient during testicular sperm retrieval [28].

Sperm retrieval success rates in patients with NOA range from 42–62% and appear to be related to the method of retrieval. Earlier research has shown that isolated regions of spermatogenic tissue may exist in testes of men with NOA [29,30]. Currently, the location of TESE biopsies are chosen arbitrarily, and as a consequence, a large portion of the biopsies yield negative results [31].

In men with NOA, previous studies have shown that testicular structure, including testicular blood flow is severely altered and strongly modified, showing decreased or absent intratesticular arterial flow compared to normal testes. In contrast, men with obstructive azoospermia (OA) exhibit uniform flow, compared to the controls. Previously it has been reported that in young boys, testicular blood flow is correlated to testicular volume, and that flow increases when the maturation process leading to spermatogenesis appears, thus suggesting a relationship between blood flow and testicular tubal function [32]. Furthermore, spermatogenesis is not uniform throughout the testis. Studies of patients with NOA showed that sperm quality was highest in areas with high sperm perfusion. Herwig et al. [33] reported in their study that indeed high levels of perfusion matched with a qualitatively and quantitatively high level of sperm retrieval from TESE. Additionally, researchers developed a method of non-invasive testicular screening using Doppler US that possesses low sensitivity (47.35%) but a high specificity (89.8%), suggesting that their technique would better predict the absence of spermatozoa than their presence, which would still favour the use of Doppler US to avoid areas of absent spermatogenesis [31]. Therefore, Doppler US could be an advantageous technique compared to standard of care in locating foci of high perfusion and assumed spermatogenesis, excluding areas of absent spermatogenesis, and subsequently improving rates of sperm retrieval in patients with NOA.

Earlier research (2001) by Belenky et al. [34] established that US-guided TESA compared to ‘blind’ TESA was a safe and accurate method for sperm retrieval in patients with NOA, but no differences were found between the two groups in pregnancy rate in the patient’s female partner. Another study in 2019 utilised contrast-enhanced US (CEUS) 10 days prior to TESA. The study assessed 70 men, 46 with NOA and the remaining with OA. The group reported that CEUS-guided TESA with cognitive fusion did not yield improved sperm retrieval outcomes of TESA in patients with NOA, potentially due to imprecise correlation between biopsy sites and main perfusion areas analysed by CEUS [35]. Conversely, a 2018 study that utilised CEUS in 120 men with NOA and subsequent micro-TESE, demonstrated improved success rates for micro-TESE potentially due to CEUS’s ability to locate the best perfusion areas. This work suggests that CEUS can highlight microvascular distribution in testicles, aid in locating areas of best perfusion over the maximal longitudinal section, and improve success rates [36]. Lastly, work by Herwig et al. [37] with patients with azoospermia undergoing TESE biopsy for retrieval of sperm showed high sperm quality in areas of high tissue perfusion. Furthermore, their results correlated the number of motile sperm isolated from tissue samples with the intensity of tissue perfusion using colour Doppler US.

Whilst showing much promise, the strength of any prognostic tool is truly known only with external validation and some limitations exist to the use of US for sperm retrieval. For US, outcomes are highly dependent on the skills of the operator. For example, Nariyoshi et al. [38] included patients from two separate clinics in Japan in their research with US-guided sperm retrievals. This could have allowed a variety of factors to potentially impact the results. In addition, successful examination and interpretation of the testicles requires training and experience. Furthermore, in examining testicular perfusion with Doppler US, previous studies were only able to locate main arteries in the testes and were unable to resolve the microvasculature. Despite this, high-density microvasculature greatly contributes to high blood testicular perfusion and this was correlated with focal spermatogenesis [31]. Nonetheless, whilst some limitations exist, Doppler US can be a potentially effective tool in aiding sperm retrieval procedures.

### Artificial intelligence

Identification of sperm parameters, selection, and assortment are an essential task when processing human testicular samples for cryopreservation or IVF. The quality of spermatozoa is one of the most important parameters for oocyte fertilisation and embryo quality. Studies have shown that abnormalities in the quality of spermatozoa correlate with cleaving embryo morphology at later stages [39].

Problems in sperm maturation cause abnormalities in sperm morphology, which need to be identified to ensure proper egg fertilisation. Assessment of sperm parameters such as semen pH, sperm morphology, viscosity, concentration, and motility can help determine male factor infertility. While some of these factors can be consistently and objectively assessed, manual assessment of other factors such as sperm morphology and motility are subjective, operator dependent, and error prone. Development of standardised and automated methods is vital for accurate and consistent results.

AI is a large ‘umbrella’ term that encompasses methods that mimic the intelligence or behavioural patterns of humans or any other living entity. Machine learning is a technique by which a computer can ‘learn’ from data, without using a complex set of different rules. This approach is mainly based on training a model from datasets. Further on, ‘deep learning’, a revolutionary method pioneered in 2012 by George E. Dahl, is a technique to perform machine learning inspired by our brain’s own network of neurones.

Artificial neural networks (or ANNs), are biologically inspired computational models developed to simulate the way in which the human brain processes data [40]. The network operates based on interconnected virtual neurones that can accept input features and produce an output decision on the basis of its ‘existence’. These networks are capable of learning under certain training instructions, generally without task-specific instructions and can make decisions on the basis of this experience. For example, in image recognition, such techniques might learn to identify images that contain spermatozoa by analysing example images that have been manually labelled as ‘spermatozoa’ or ‘no spermatozoa’ and using analytical results to identify spermatozoa in other images. Such ANNs have been shown to classify and recognise patterns accurately [41].

Built on previous concepts of neural networks, deep neural networks (DNN) are ANNs with multiple layers between the input and output layers [42]. DNNs can model complex non-linear relationships. For example, a DNN that is trained to recognise histological cell types will study the given image and calculates the probability that the cell in the image is of certain type. The user can review the results and select which probabilities the network should display and return the proposed label. Each mathematical manipulation as such is considered a layer, a complex DNN, may have many layers, lending itself the name ‘deep’ networks.

When addressing male infertility, the main challenges are prediction of sperm presence, identification of sperm on biopsy extraction, and qualification of sperm integrity after extraction. Currently, these processes remain unaided by AI systems, are not automated, and are operator dependent.

One fundamental advantage of ANN exists in its ability to predict outcomes based on previous data. In a retrospective analysis of data collected from physical examinations Samli and Dogan [43] developed an ANN for predicting spermatozoa prior to testicular biopsy in men with NOA and compared it to a standard logistic regression model. Using factors such as age, duration of infertility, serum hormone levels, and testicular volumes, the group was successful in creating a model with a significantly higher sensitivity than the logistic regression model and was able to correctly predict outcomes and achieve clinically-acceptable sensitivity in 59 of 73 patients in the test set.

An alternative method to assess the probability of finding sperm was used by Ramasamy et al. [44]. Their method consisted of a retrospective analysis of men who underwent micro-TESE. Rather than training ANN with images their method consisted of using readily available clinical features to model and predict the chance of identifying sperm with micro-TESE in men with NOA. The proposed model demonstrated 59.4% success in correctly predicting the outcome of sperm retrieval based on pre-existing clinical evidence. Although showing promising data, these results were not generalisable and other studies are required for external validation.

In the field of reproductive medicine, there are no existing computer-aided sperm analysis systems for testicular biopsies. This process is very operator dependent and relies on manual image analysis for sperm identification. To tackle this problem, Wu et al. [45] proposed a deep convolution neural network method in which a dataset of 702 de-identified images from testicular biopsies were collected from testicular biopsies of 30 patients. The group was able to achieve a mean average precision of 0.741 with an average recall of 0.376 on their dataset, suggesting that deep learning is an efficient method of finding sperm in testicular biopsy samples.

In a clinical setting, ANNs, and deep learning methods hold true potential for innovation in automatic assessment of human sperm due to the ability to work with low resolution images and unstained sperms, in real time and with high accuracy. Javadi and Mirroshandel [39] suggested a deep learning method for selecting the best sperms in an ICSI procedure. The proposed model extracts features of the acrosome, head shape, and vacuole from sperm images gathered in real time. The method was able to select the best fresh sperm for injection, and ultimately achieved a better accuracy than existing state-of-the-art methods in acrosome and vacuole abnormality detection on the proposed benchmark. Experimental results showed high accuracy of the proposed deep learning model.

Similarly, work by McCallum et al. [46] focussed on assessing the quality of sperm DNA using deep learning-based methods. Traditionally, sperm quality has been assessed by skilled clinicians to select the best sperm based on various morphological and motility criteria, but without direct knowledge of their DNA cargo. The group developed a DNN approach that is directly compatible with current, manual microscopy-based sperm selection and complementary to current clinical selection. Overall, the team was able to rapidly predict DNA quality (<10 ms/cell) and sperm selection within the 86th percentile from a given sample.

Motility estimation is another essential step in evaluation of male fertility. Because it can be considered as a functional test, it is a direct measurement of the energy status of the mammalian sperm. An AI-assisted method of evaluating sperm motility could thus be another beneficial tool for clinicians to select sperm after retrieval. To address this task, Contri et al. [40] focussed on ANNs for the definition of kinetic subpopulations and epididymal spermatozoa in domestic cats. This study prospectively collected electro-ejaculated samples from seven adult cats. The motility pattern of the feline semen was evaluated using a computer-assisted sperm analyser (CASA) system IVOS 12.3 (Hamilton-Thorne Bioscience, Beverly, MA, USA). The results of their study demonstrated the ability of ANNs to differentiate significant kinetic differences in electro-ejaculated vs epididymal samples.

The role of AI, neural networks, and deep learning, in the realm of fertility and reproduction still remains to be determined. While some technologies have shown promise, to our knowledge, studies have not yet determined the best ways to use AI for sperm extraction and reproduction. However, several have reported the use of ANN in medicine, mainly for the diagnosis and prognostic evaluation of several pathologies [41,43]. For example, work by Berlin et al. [47] has demonstrated the ability of machine learning to improve the efficiency and consistency of the automated planning method for prostate volumetric arc radiation therapy. Recently, the field of ophthalmology established itself as a paradigm shifter in the use of clinical AI. The IDx-DR (Digital Diagnostics, formerly IDx) is an autonomous AI designed to detect diabetic retinopathy and diabetic macular oedema. In 2018, it became the first United States Food and Drug Administration (FDA)-approved autonomous AI in any field of medicine.

Nonetheless, the use of AI, to tackle problems of sperm identification is a challenging task. For example, the number of sperm images available in real time can be a limiting factor for the AI training phase. The normal and abnormal sperm classes are highly imbalanced, thus making the problem harder. Furthermore, the images that are available in real time are taken using a low-magnification microscope and the details of these images are not clear, the pictures are very ‘noisy’ [39]. Overall, while some barriers exist to the use of AI and deep learning in reproductive medicine, overcoming these barriers will allow rapid predicting capabilities, identification mechanisms, and analysis of sperm integrity done in real time, without the need of samples being stained for identification purposes (Table 2) [4,7,10,11,13,14,17–24,28,31,33,36–39,41,43].

**Table 2.** Advantages and disadvantages of different retrieval/identification methods

Retrieval/identification method	Advantages	Disadvantages
Micro-TESE	Good chance of sperm recovery [4]	Risk of damage to testis architecture [7] Success may depend on surgical skill [7]
Multiphoton microscopy	3D *in vivo* histological images Depth of penetration up to 400 µm Real-time analysis [10] Lower energy laser = minimal damage [11] Decreased operating time	Human studies not conducted to determine risk of genetic damage [13]
Raman spectroscopy	Flexible probe for surgical ease Non-destructive near infrared light source [14]	Safety not assessed in human models Specimen preparation takes 2 minutes and can be influenced by light pollution [17]
Full-field optical coherence tomography	Fast and easy to obtain images [18] Safe light source from 150-W halogen lamp [19]	Absence of cellular details Limited depth of imaging below cellular surface [20] Only images *ex vivo* specimens [20] Efficacy not proven compared to other techniques [19]
ORBEye	Elimination of eyepieces allows for better surgical posture [21] Simple to use and easy to transport May allow for shorter surgery times [21] Wider FOV and longer DOF eliminates need for frequent repositioning [22] 3D viewing monitors provides optimal teaching/demonstration environment [23]	Cost Surgical assistants have rotated surgical view, which can lead to confusion during operation [24]
Ultrasonography	Fast, easy, portable [28] Widely available [28] Ability to locate areas of high perfusion [33] Help rule out areas of absent spermatogenesis [31] Improve success rates of sperm retrievals [36,37]	Operator dependent [38] Requires training and experience [38] Difficulty with resolving microvasculature of the testicle [31]
Artificial Intelligence	Automated assessment of extracted sperm [37,38,44] Real-time analysis of sperm [37] Ability to work with unstained images [37] High accuracy [37] Ability to predict outcomes prior to extraction [41] Avoid operator dependence [43]	Training phase of AI can be limited by the number of sperm images available [39] Limited by quality of microscopy images [39] Technology is not widely available Requires collaboration between computer scientists and clinicians

FOV: field of view; DOF, depth of field.

## Conclusion

Multiple new promising technologies have emerged recently to assist urologists during sperm retrieval for a male with infertility. The use of MPM, RS and FFOCT during a micro-TESE procedure can help distinguish tubules with and without spermatogenesis, a role that can also be potentially played by Doppler US. ORBEYE technology can be used as a valid alternative to the traditional microscopy technique. Finally, some studies have also shown promising results for the use of AI and neural networks to enhance sperm identification in surgically extracted sperm samples.
